# Spatial distribution of planktonic bacterial and archaeal communities in the upper section of the tidal reach in Yangtze River

**DOI:** 10.1038/srep39147

**Published:** 2016-12-14

**Authors:** Limin Fan, Chao Song, Shunlong Meng, Liping Qiu, Yao Zheng, Wei Wu, Jianhong Qu, Dandan Li, Cong Zhang, Gengdong Hu, Jiazhang Chen

**Affiliations:** 1 Freshwater Fisheries Research Center, Chinese Academy of Fishery Sciences, Scientific Observing and Experimental Station of Fishery Resources and Environment in the Lower Reaches of the Yangtze River, Wuxi 214081, China

## Abstract

Bacterioplankton and archaeaplankton communities play key roles in the biogeochemical processes of water, and they may be affected by many factors. In this study, we used high-throughput 16S rRNA gene sequencing to profile planktonic bacterial and archaeal community compositions in the upper section of the tidal reach in Yangtze River. We found that the predominant bacterial phyla in this river section were Proteobacteria, Firmicutes, and Actinobacteria, whereas the predominant archaeal classes were Halobacteria, Methanomicrobia, and unclassified Euryarchaeota. Additionally, the bacterial and archaeal community compositions, richnesses, functional profiles, and ordinations were affected by the spatial heterogeneity related to the concentration changes of sulphate or nitrate. Notably, the bacterial community was more sensitive than the archaeal community to changes in the spatial characteristics of this river section. These findings provide important insights into the distributions of bacterial and archaeal communities in natural water habitats.

Microorganisms present in water are key players in biogeochemical processes (e.g., nitrogen, sulphur, and carbon cycling) and are primary producers and consumers in these ecosystems. Furthermore, these microorganisms are major players in processes controlling water quality, particularly processes mediating the fate of pollution released to the water environment^1^. Although planktonic bacteria and archaea are the two most important groups of microorganisms in water, they have diverse evolutionary histories. Bacteria have long been recognised as having broad distributions throughout all environments around the globe. In contrast, archaea were once thought to inhabit primarily extreme environments, such as psychrophilic^2^ and hypersaline^3^ environments, deep-sea hydrothermal vents^4^, and hot springs^5^. However, with the emergence and application of modern molecular techniques, researchers have shown that both bacteria and archaea are widely distributed in many diverse environments^6^. Additionally, nonextremophilic archaea may also be important players in extant global biogeochemical cycles^7^, similar to bacteria. In addition, owing to the potential differences in the functional division of bacteria and archaea based on their diversities in the same environment^8^, these organisms may show different responses to a variety of habitats. Analysis of the responses of these organisms is critical for improving our understanding of the mass and energy fluxes of ecosystems.

The tidal reach area of a river is a complex habitat that can be affected by runoff, tide, and seasonal variations. Although the upper section of tidal reach can refer to the area from the tidal current limit to the tidal limit, where the tide had not arrived, the river can also be affected by the tidal wave. In one study, the tidal limit of Yangtze River was found to be located between Anqing (in the dry season) and Nanjing (in the wet season); however, the tidal current limit is located between Zhenjiang and Jiangxinsha (upstream of the Jingjiang location)^9^. In this area, the water-surface slope and natural flowing velocity are affected by the tidal wave; this influence decreases with the gradual weakening of the tidal wave from the tidal current limit to the tidal limit. Moreover, this effect could also modify the river bed in this river section by changing the natural conditions of sedimentation and erosion. Additionally, after the construction of the Three Gorges Dam (the largest hydropower station in the world) in mid-2006, the conditions of the tidal area have become even more complicated. The natural flowing velocity and natural conditions of sedimentation and erosion may be altered further owing to the combined effects of all factors, leading to distinct microbial communities within this habitat. Studies of changes in these microbial communities will improve our understanding of aquatic microbial ecology.

Many studies describing microbial communities have focused on water environments, such as estuaries^10^,^11^,^12^, rivers^13^,^14^, lakes^15^,^16^,^17^, and oceans^18^,^19^. Although researchers have also evaluated bacterioplankton communities within these water environments, few studies of planktonic archaeal communities in freshwater habitats have been performed, particularly in fluvial ecosystems^20^. Some other studies^21^,^22^,^23^,^24^ have reported variations in bacterioplankton and archaeaplankton communities across freshwater and ocean environments, demonstrating that spatial variability was greater than seasonal patterns of variation. In addition to habitat heterogeneity^25^, planktonic bacterial and archaeal communities can also respond to changes in many environmental factors in water, such as salinity^26^,^27^, dissolved organic matter (DOM)^28^, alkalinity^29^, temperature, and soluble reactive phosphorus (SRP)^20^. Moreover, temporal variation in the microbial community has been shown to be greater than spatial variation in the same habitat^15^,^30^,^31^. In the upper section of the tidal reach in Yangtze River, inherent spatial heterogeneity may exist due to the tidal wave, dynamic changes in the river itself, and artificial engineering, which may result in regular patterns of spatial distributions of bacterial and archaeal communities. However, no studies have yet examined these factors with high-depth coverage or made comparisons of the spatial distributions of archaeal and bacterial communities in this water environment.

Accordingly, in this study, we aimed to explore the bacterial and archaeal community structure compositions in this important water area in the Yangtze River and to investigate the response patterns of these microorganisms to the inherent spatial heterogeneity of the ecosystem. We established sampling sites between the Anqing and Jingjiang cities located in Anhui and Jiangsu provinces of China respectively, stretching across more than 400 km; this region included many economically developed cities, which could have affected the microbial community compositions and distributions. Our findings provide important insights into the distributions of bacterial and archaeal communities in natural water habitats.

## Results

### Community structure, richness, and diversity of planktonic bacteria and archaea

After pyrosequencing and filtering operations, a total of 908,735 valid reads (465,311 for bacteria and 443,424 for archaea) and 3, 714 operational taxonomic units (OTUs; 987 for bacteria and 2,727 for archaea) were obtained from all water samples. All bacterial OTUs were assigned to 30 different phyla or groups, 76 classes or groups, and 377 genera or groups. The archaeal OTUs were assigned to four different phyla or groups, 23 classes or groups, and 80 genera or groups.

**Figure 1:**
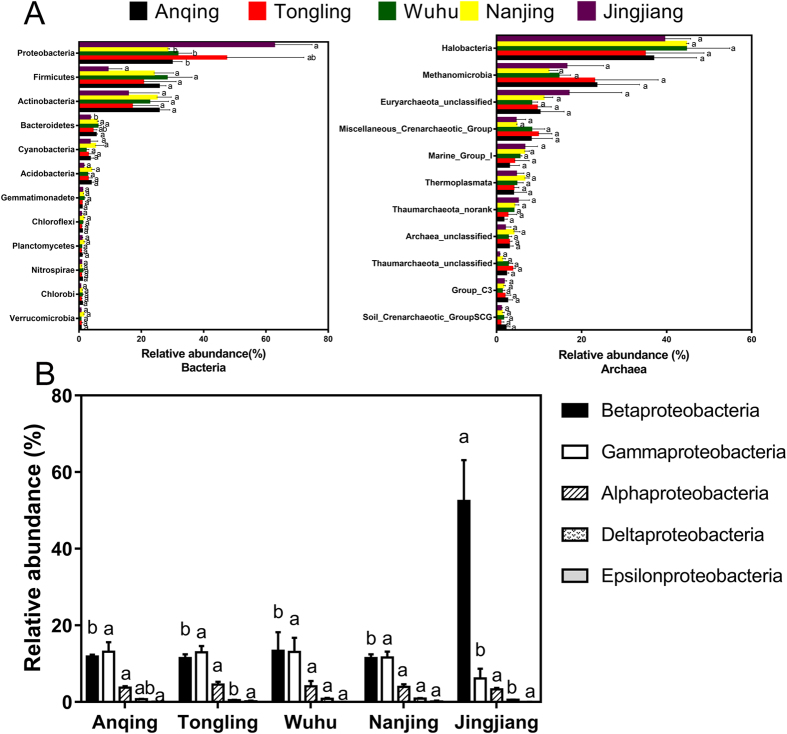
Comparison of the taxonomic composition in all water samples along the lower reaches of Yangze River. The relative abundances (percentage) at phylum (bacteria) and class levels (archaea) that were more than 1% were listed in panel A. The relative abundances of the five classes within the Proteobacteria phylum were listed in panel B. The legends with different colors in panel A represented the five sampling sites, while the different filling patterns of them in panel B represented different classes within the Proteobacteria phylum. The letters upon at the right side of the columns show the differences between groups. The same letters mean no significant differences, while different letters mean that there are statistically significant differences between groups at 95% level.

Among the 30 phyla or groups, Proteobacteria (39.95% ± 17.35% [mean ± standard deviation]), Firmicutes (21.54% ± 9.07%), and Actinobacteria (21.17% ± 7.25%) were the three predominant planktonic bacterial phyla identified in the upper section of the tidal reach of Yangtze River (Fig. 1A). The other three phyla, Bacteroidetes (5.01% ± 1.22%), Cyanobacteria (3.45% ± 1.97%), and Acidobacteria (2.93% ± 1.23%) also accounted for a relatively large proportion of the identified organisms. An additional comparison of five sampling sites showed that the bacterial composition of the Jingjiang location was significantly different from the other four locations at the phylum level. The relative mean abundance of Proteobacteria was highest in Jingjiang (*p* < 0.05), while the relative mean abundances of Firmicutes, Actinobacteria, Bacteroidetes (*p* < 0.05), and Acidobacteria (*p* < 0.05) were lowest at this location. Moreover, the abundance of Proteobacteria was negatively correlated with the geographical distances between the sampling sites and mouth of the estuary (*p* < 0.05).

Although the classes within the phylum Proteobacteria generally play a key role in the biogeochemical cycles of water ecosystems, we focused on the distributions of these groups in this specific river section (Fig. 1B). The results showed that the two most abundant classes were Betaproteobacteria (23.61% ± 19.30%) and Gammaproteobacteria (11.37% ± 3.49%). Additionally, Alphaproteobacteria (3.91% ± 0.82%), Deltaproteobacteria (0.69% ± 0.20%), and Epsilonproteobacteria (0.09% ± 0.13%) were also present in this river section. Comparisons of these classes among the five sampling sites revealed that Betaproteobacteria and Gammaproteobacteria showed significantly higher and lower abundance, respectively, at Jingjiang than at any other site (*p* < 0.05). These classes were also correlated negatively and positively, respectively, with the geographical distances between the sampling sites and the mouth of the estuary (*p* = 0.001, R^2^ = 0.614; *p* = 0.003, R^2^ = 0.499).

The four phyla or groups of planktonic archaea in this river section were Euryarchaeota (74.75% ± 4.96%), Thaumarchaeota (21.79% ± 4.33%), unclassified Archaea (2.91% ± 1.17%), and Crenarchaeota (0.55% ± 1.02%). The main communities of planktonic archaea at the class level are listed in Fig. 1A. The three predominant classes or groups were Halobacteria (40.07% ± 8.92%), Methanomicrobia (17.94% ± 9.04%), and unclassified Euryarchaeota (11.17% ± 6.28%). Additionally, the classes of miscellaneous Crenarchaeotic group (7.03% ± 3.47%), marine group I (5.12% ± 2.49%), Thermoplasmata (4.74% ± 1.89%), and unranked Thaumarchaeota (3.48% ± 1.88%) also covered a large proportion of the identified organisms. The relative mean abundance of unclassified Euryarchaeota, marine group I, and unranked Thaumarchaeota was highest at the Jingjiang location. Additionally, the relative mean abundances of marine group I (*p* = 0.047, R^2^ = 0.271) and unranked Thaumarchaeota (*p* = 0.014, R^2^ = 0.381) were negatively correlated with the distances between the sampling sites and the mouth of the estuary.

The richnesses of bacterial and archaeal communities were estimated by the Chao index, and the diversities of these communities were determining using the Shannon index (Table 1). We found that the Chao index value of the archaeal community at the Jingjiang location was significantly higher than that at other locations (*p* < 0.05). Additionally, regression analysis showed that the Chao index value of the archaeal community (*p* = 0.001, R^2^ = 0.588) was affected by the distances between the sampling sites and the mouth of the estuary. The Chao index value of the bacterial community in this river section was also negatively correlated with the distances between the sampling sites and the mouth of the estuary (*p* = 0.044, R^2^ = 0.276) to some extent.

### Functional diversities in bacterial and archaeal communities

**Figure 2:**
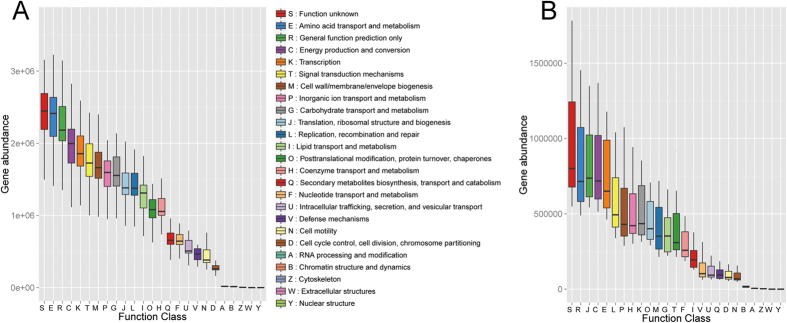
Predictive functional profiling of microbial communities using 16S rRNA marker gene sequences. A represented the function classes of bacteria and their gene abundance, while B represented that of archaea and their gene abundance.

Bacterial and archaeal functional community profiles were examined, and their corresponding gene abundances are shown in Fig. 2A and B. We found that the gene abundances of all predicted functional groups in the bacterial community were all higher than those in the archaeal community by more than an order of magnitude. Additionally, there were significant differences in relative gene abundance for some main functional classifications between bacterial and archaeal communities; for example, the functions of “translation, ribosomal structure, and biogenesis”, “replication, recombination, and repair”, and “coenzyne transport and metabolism” were obviously increased in the archaeal community (relative abundances: 9.30% ± 1.08%, 6.41% ± 0.39%, and 5.67% ± 0.57%, respectively) compared with those in the bacterial community (relative abundances: 5.27% ± 0.17%, 5.20% ± 0.28%, and 4.04% ± 0.18%, respectively).

These findings demonstrated that the richness of an archaeal community could be affected by the distances between the sampling sites and the mouth of the estuary. Therefore, we next determined whether the functions of the bacterial and archaeal communities could be affected by this distance. Regression analysis results (Table 2) showed that most of the bacterial community function could be affected by the distances between the sampling sites and the mouth of the estuary. In contrast, only the archaeal community function of cytoskeleton (Z) could be affected by this parameter. The abundances of the OTUs corresponding to all functions tended to decrease as the distance from the mouth of the estuary increased.

### Ordination of bacterial and archaeal communities and potential influencing factors

**Figure 3:**
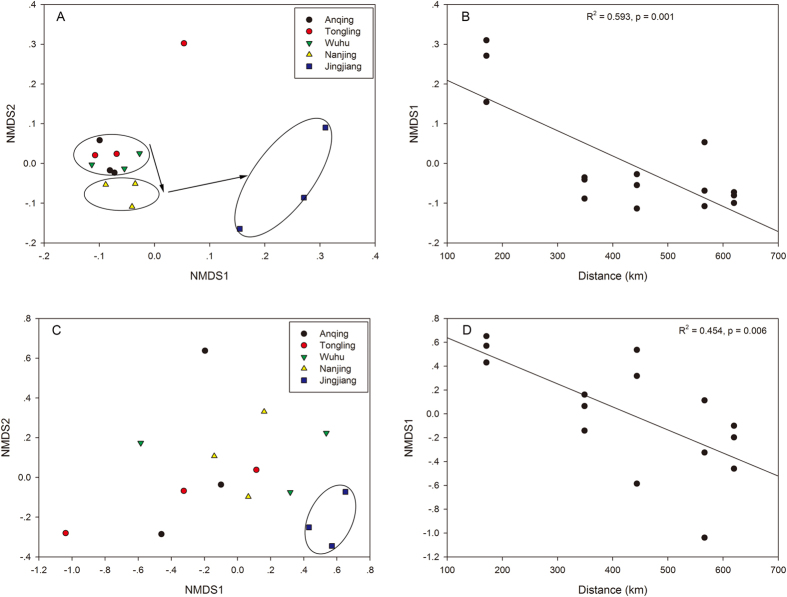
(**A**,**C**) NMDS plots of the first and second NMDS dimensions relating bacterial and archaeal community compositions respectively between sampling sites. Symbols representing communities present in each sampling site are as follows: Anqing, Tongling, Wuhu, Nanjing and Jingjiang. Percentages given in axes labels are the % variation in community composition explained by the respective NMDS dimensions. (**B**,**D**) Linear regression of the distances between sampling sites and mouth of estuary versus dimension 1 of the NMDS analysis. The strength of the linear relationship is indicated by the regression coefficient (R^2^) on the plot.

A nonmetric multidimensional scaling (NMDS) diagram of 15 samples based on Bray-Curtis similarity values of the bacterial community (Fig. 3A) showed that all samples were separated into three groups. Among these groups, most of the samples belonged to three sites in the upper regions of the area, i.e., Anqing, Tongling, and Wuhu, while samples from Nanjing and Jingjiang were distinct and distributed along the x-axis (the first NMDS dimension). The NMDS diagram of samples based on Bray-Curtis similarity values for the archaeal community (Fig. 3C) showed that only the group of samples from Jingjiang was distinct from the other samples, tending to be distributed along the x-axis. Thus, the ordinations of bacterial and archaeal communities were also somewhat related to the distances from the mouth of the estuary (Dongwangsha).

The distances from Dongwangsha were then selected as predictor variables, and the first NMDS dimensions of bacterial and archaeal similarities were set as the outcome variables for linear regression analysis. The results showed that the first dimension of the NMDS analysis of the bacterial community composition plotted against the distances indicated a predictable community response (R^2^ = 0.593 and *p* = 0.001; Fig. 3B), suggesting that the bacterial community could be affected by the distance from the mouth of the estuary. At the same time, the first dimension of NMDS analysis of archaeal community composition plotted against the distances also indicated a predictable community response (R^2^ = 0.454 and *p* = 0.006; Fig. 3D), suggesting that the archaeal community was also closely influenced by the distances from Dongwangsha. However, most of the communities could not be separated distinctly.

In order to investigate the additional physicochemical factors influencing the bacterial and archaeal communities in the upper section of the tidal reach of Yangtze River, physicochemical parameters, such as salinity, temperature, total nitrogen, total phosphorus, permanganate index, chlorophyll a, ammonia, nitrate, nitrite, cadmium, copper, lead, zinc, mercury, arsenic, oil, turbidity, fluorine, chlorine, sulphate, bromine, total organic carbon (TOC), total carbon (TC), and inorganic carbon (IC), were detected. However, because salinity could not be detected with the SYC1–2 salinity meter, it was excluded, and only the other physicochemical parameters were selected as outcome variables, with the distance from Dongwangsha set as the predictor variable for linear regression analysis (Table 3). The results showed that the distances from Dongwangsha were positively correlated with ammonia but negatively correlated with nitrate, sulphate, cadmium, lead, turbidity, chlorine, and bromine. Furthermore, we then performed regression analysis using these eight physicochemical parameters as the predictor variables and the NMDS1 values of the bacterial and archaeal communities as the outcome variables (Table 3). The results showed that among the eight parameters, nitrate and sulphate could significantly affect the distribution of the bacterial community, whereas only nitrate could significantly affect the distribution of the archaeal community. These parameters all exhibited negative correlations with the first dimensions of NMDS values of bacterial and archaeal communities.

### Specific bacterial and archaeal taxa analyses

**Figure 4:**
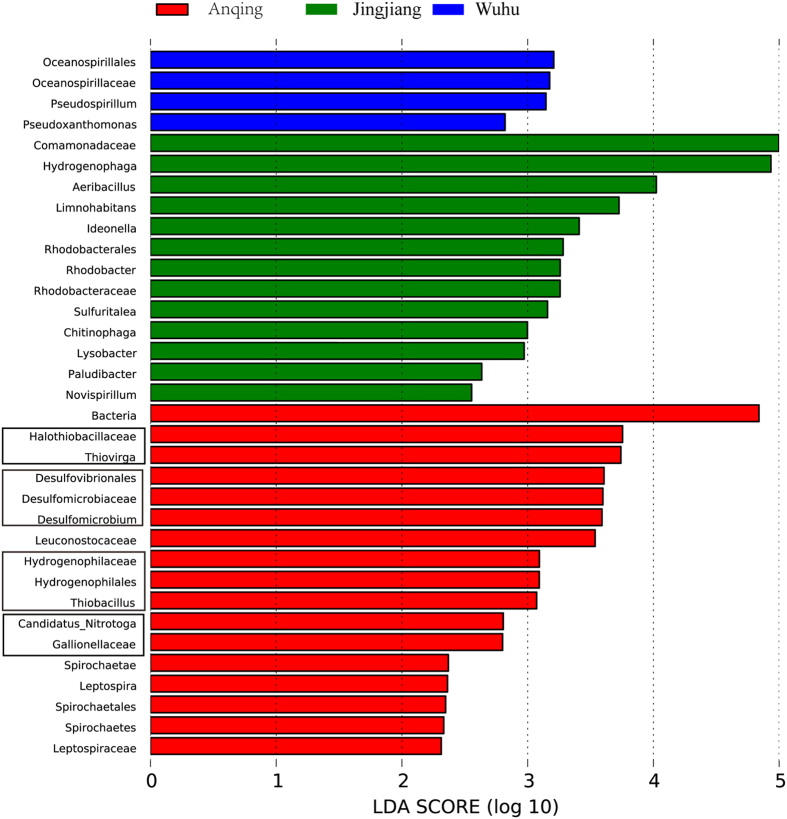
LEfSe identified the most differentially abundant taxa in specific sampling sites. The red, green and blue colors represented bacterial communities of Anqing, Jingjiang and Wuhu respectively. Only taxa meeting an LDA significant threshold >2 were shown in the figure. The taxa in the same rectangles that were related to sulfur and nitrogen recycles were subordinate relationships.

From the above studies, we found that the factor of distance from Dongwangsha may affect the bacterial and archaeal communities in the upper section of the tidal reach of Yangtze River by altering the concentrations of sulphate and nitrate in the water. Thus, we next aimed to obtain some specific bacterial and archaeal taxa from the various sampling sites and investigate whether these taxa were related to the distance from Dongwangsha and the two parameters identified above. For the archaeal community, only the Jingjiang location was specific for the genus *Methanothermobacter*. Three specific sampling sites and their specific bacterial taxa are shown in Fig. 4. Analysis of the specific bacterial taxa at Anqing showed that most organisms were related to recycling of sulphur (the family *Halothiobacillaceae* and subordinate genus *Thiovirga*; the order *Hydrogenophilales*, subordinate family *Hydrogenophilaceae*, and subordinate genus *Thiobacillus*; the order *Desulfovibrionales*, subordinate family *Desulfomicrobiaceae*, and subordinate genus *Desulfomicrobium*) and nitrogen (the family *Gallionellaceae* and subordinate genus *Candidatus_Nitrotoga*). Notably, nearly all taxa in subordinate relationships exhibited the same abundances. Next, the taxa with the lowest taxonomic levels (e.g., the genera *Thiovirga, Thiobacillus, Desulfomicrobium*, and *Candidatus_Nitrotoga*) were selected to investigate their relationships with the distance from Dongwangsha. The genus *Desulfomicrobium* was only present at the Anqing location, while the other three genera were all positively correlated with the distances between sampling sites and the mouth of the estuary (*Thiovirga: p* = 0.001, R^2^ = 0.594; *Thiobacillus: p* = 0.008, R^2^ = 0.429; *Candidatus_Nitrotoga: p* = 0.018, R^2^ = 0.358).

## Discussion

In this study, we reported the first high-depth coverage taxonomic survey of planktonic bacterial and archaeal communities in the upper section of the tidal reach of Yangtze River and the response patterns of these communities to the inherent spatial heterogeneity of the region.

Our findings showed that the predominant bacterial communities in this river section were Proteobacteria, Firmicutes, and Actinobacteria, which were all once abundant in many of the freshwater lakes and reservoirs in China^32^ and exhibited high abundances in some other freshwater environments^30^,^33^,^34^,^35^. From these findings, we found that the bacterial communities in this section of Yangtze River exhibited a typical feature of freshwater populations. Additionally, although the bacterial community at the Jingjiang location, which was the nearest to the mouth of the estuary, was significantly different from that in other locations, the beta-Proteobacteria class, which is thought to be distributed primarily in freshwaters^36^,^37^, showed the highest relative abundance at this location. Thus, we concluded that the freshwater bacterial community structure in this river section was consistent with the results showing lack of salinity and with a previous report showing that this river section was within the upper reaches of the tidal current limit^9^. However, we also found that the predominant phylum of Proteobacteria was negatively correlated with the geographical distances between the sampling sites and the mouth of the estuary, indicating that the bacterial community compositions of this river section may be affected by some factors induced by inherent spatial heterogeneity.

Although bacterial communities have been studied extensively, studies of the archaeaplankton community in freshwater are relatively scarce. Nevertheless, previous studies have shown that the dominant archaeal communities were significantly diverse, even at the phylum level. The predominant archaeaplankton phylum in this river section was Euryarchaeota, which was consistent with the results of a study in the Jiulong River Watershed^20^ but different from the results of a study of the freshwater area of Pearl River^24^. The predominant phylum in the freshwater area of Pearl River was Thaumarchaeota, similar to that of Yellowstone Lake^38^. Additionally, the results of studies on planktonic archaeal communities in Taihu Lake in China and Vilar Lake in Spain^15^,^39^ showed that the dominant phyla were Crenarchaeota, Euryarchaeota, and Parvarchaeota (in decreasing order) for Taihu Lake and Crenarchaeota and Euryarchaeota (in decreasing order) for Vilar Lake.

The regression analysis in the present study showed that among the 11 main classes, only the relative abundances of marine group I and unranked Thaumarchaeota were negatively correlated with the distances between the sampling sites and the mouth of the estuary, similar to the response patterns of some bacterial populations. Additionally, the predominant bacterial phylum of Proteobacteria was negatively correlated with the geographical distances between the sampling sites and the mouth of the estuary, whereas only the classes of marine group I and unranked Thaumarchaeota, whose relative abundances were low, showed the same trend, indicating that the planktonic archaeal community was somewhat less sensitive to the changes in inherent heterogeneity associated with geographic distance. This finding was further supported by our functional diversity analysis. Moreover, our regression analysis showed that most of the function of the bacterial community was affected by the distances between the sampling sites and the mouth of the estuary; in contrast, only the archaeal function of cytoskeleton was affected. These findings may also be supported by analysis of the specific bacterial and archaeal taxa, where more sampling sites existed for more specific bacterial taxa than for archaeal taxa. Functional diversity analysis also showed that the gene abundances of all predictive functional groups in the bacterial community were higher than those in the archaeal community, suggesting that the bacterial community played a greater role than the archaeal community in this river section.

Interestingly, we found that Chao index values but not the Shannon index values of the bacterial and archaeal communities were negatively correlated with the geographical distances between the sampling sites and the mouth of estuary, suggesting that the effects may have occurred from a region below the river section. It is possible that some more adaptable marine bacterial and archaeal taxa may disperse, causing changes in the richness of the bacterial and archaeal communities in the upper section of the tidal reach of Yangtze River. However, these changes were not great enough to also cause changes in diversity.

Ordination analysis of bacterial and archaeal communities also provided evidence of the differences between sampling sites, where more sample groups from the same sites could be separated based on bacterial communities. This confirmed our results showing that the bacterial community was more sensitive than the archaeal community to the distances from the mouth of the estuary in the upper section of the tidal reach of Yangtze River.

Linear regression analysis indicated that the distance from the mouth of the estuary may affect the bacterial and archaeal communities by altering the concentrations of sulphate and nitrate. Additionally, evaluation of the linear discriminant analysis effect size (LEfSe) showed that the Anqing location was specific for bacterial groups related to sulphur and nitrogen recycling. Accordingly, the abundances of these groups were positively correlated with the distance from the mouth of the estuary, supporting our above-mentioned conclusions. Moreover, the concentrations of sulphate and nitrate may be negatively correlated with the distance from the estuary owing to the accumulation of pollutants from cities along the riverside^40^. Thus, the spatial heterogeneity which made the structures of bacterioplankton and archaeaplankton communities all present some linear distribution features in the upper section of the tidal reach in Yangtze River was closely related to the concentration changes of sulphate and nitrate caused by the accumulation of pollutants from cities along the riverside. Nevertheless, some other parameters might also be factors affecting the spatial heterogeneity in this river section. Salinity, in particular, which was not detected in this study, might show slight changes along this river section, and affect the distribution of some bacterial and archaeal species in this river section. And because the tidal limit and tidal current limit are affected by the energy of the tide^41^, runoff, and topographical features of the river^42^, these factors are also likely to affect the spatial heterogeneity of this river section. Then, the responses of bacterial and archaeal communities in the upper section of the tidal reach of Yangtze River to variations in geographic distances from the mouth of the estuary could also be attributed to the mixed effects of the factors above. Importantly, additional physicochemical parameters not examined in this study may also be involved.

In summary, our results showed the bacterioplankton and archaeaplankton community’s compositions in the upper section of tidal reach in Yangze River generally presented the characteristics of their freshwater populations. However, the distributions of them in this river section were affected by spatial heterogeneity which was closely related to the concentration changes of sulphate and nitrate. In addition, the bacterioplankton community was more sensitive than the archaeaplankton community to changes in the spatial characteristics of this river section. However, owing to the fact that the physical-chemical parameters selected were not so much, then additional physicochemical parameters not examined in this study may also be involved affecting the distributions of bacterioplankton and archaeaplankton communities. In the future, if more targeted parameters are selected, and the method of metagenome sequencing is used for investigating the bacterioplankton and archaeaplankton communities in this river section, then more valuable results could be obtained.

## Methods

### Sample collection

**Figure 5:**
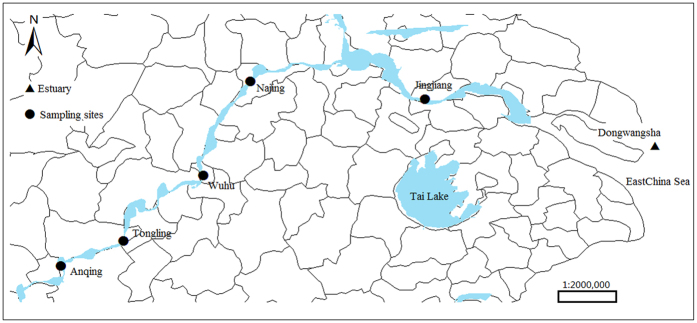
Location of the five sampling sites along the lower reaches of Yangze River. The Dongwangsha site located in the estuary area was taken as the starting point to caculate the distance between sampling sites and mouth area of the estuary. The ArcGIS 10.2 software (http://resources.arcgis.com/zh-cn/home/) was used to generate the map.

The water samples were collected from five locations in the tidal reach of Yangtze River (Fig. 5) from August 25–28, 2015. ArcGIS 10.2 software (http://resources.arcgis.com/zh-cn/home/) was used to generate the map. The geographical position information (longitude and latitude coordinates) for these locations is listed in Table S1. The Dongwangsha site (N31.35°E121.58°) located in the estuary area was considered the starting point to calculate the distance between sampling sites and the mouth area of the estuary. A river cross-section was designated in each sampling location, and three repeated samplings were performed for each section. Five hundred millilitres of water was collected at each time at a depth of 100 cm below the surface and was filtered through a 0.2-μm (47-mm diameter) pore size hydrophilic polyethersulphone membrane to obtain microbe samples for our analysis. Samples were stored at −80 °C until use. At the end of the experiment, 15 samples were obtained from sampling locations at Anqing, Tongling, Wuhu, Nanjing, and Jingjiang.

### DNA extraction, 16S rRNA gene amplification, and pyrosequencing analysis

DNA was extracted in duplicate from membrane filters using a PowerWater DNA Isolation Kit (MO BIO, USA) according to the manufacturer’s instructions. The DNA was quantified and then stored at −20 °C until further use.

Polymerase chain reaction (PCR) was used to amplify the V3–V4 hypervariable region of the bacterial 16S rRNA gene and the V3–V6 hypervariable region of the archaeal 16S rRNA gene. The primers used for bacterial 16S rRNA gene PCR amplification were 338F (5′-ACTCCTACGGGAGGCAGCAG-3′) and 806R (5′-GGACTACHVGGGTWTCTAAT-3′), and the primers for archaeal 16S rRNA gene PCR amplification were Arch344F (5′-ACGGGGYGCAGCAGGCGCGA-3′) and Arch915R (5′-GTGCTCCCCCGCCAATTCCT-3′). All primers contained barcodes for each sample^43^. The products from the three replicate amplifications of the bacterial or archaeal 16S rRNA gene were pooled and evaluated on 2% agarose gels (TBE buffer). Amplicons were purified with a DNA gel extraction kit (Axygen, China), quantified with a QuantiFluorTM-ST fluorometer (Promega, Madison, WI, USA), pooled at equimolar concentrations, and finally sequenced on an Illumina MiSeq PE300 platform at Majorbio Bio-Pharm Technology Co., Ltd. (Shanghai, China).

### Bioinformatics analysis

The acquired sequences were filtered by evaluating the data quality and removing primers and barcodes using Trimmomatic and FLASH software. The filtering steps were performed as previously described^30^. Unique sequences were clustered into OTUs with similarities greater than 97%^44^ using the Usearch program (version 7.1; http://drive5.com/uparse). Sequences were then assigned to taxa using the Ribosomal Database Project classifier^45^ compared with the SILVA bacterial and archaeal database (version 119; http://www.arb-silva.de) at a 70% confidence level. We used the method of Quantitative Insights into Microbial Ecology (QIIME) to analyse the community richness (Chao 1 estimator: http://www.mothur.org/wiki/chao) and the community diversity (Shannon index: http://www.mothur.org/wiki/Shannon). NMD) was performed using R language.

PICRUSt software (Phylogenetic Investigation of Communities by Reconstruction of Unobserved States, version 1.0.0) was used to explore the functional profiles of our bacterial and archaeal community datasets^46^. The eggNOG database (evolutionary genealogy of genes: Non-supervised Orthologous Groups, version 4.5; http://eggnog.embl.de/) was used to annotate functional information^47^. The function abundance profile was created based on the abundance of all OTUs and their corresponding COG functional annotations.

We characterised microorganisms specific to different grouping types by the LEfSe method, which evaluated both statistical significance and biological relevance^48^.

### Determination of physicochemical parameters

Total nitrogen and total phosphorus were measured using the alkaline potassium persulphate oxidation method. The potassium permanganate index (COD) was measured using the acidic potassium permanganate oxidation method. Cholorophyll a was measured with the ethanol extraction spectrophotometric method. Nessler colorimetric assays were used to measure the concentration of ammonia in water. The concentration of nitrite in water was measured with a DIONEX ICS 3000 Ion Chromatograph. The cadmium, copper, lead, zinc, and arsenic levels in water were measured with a Beifen-Ruili WFX-210 atomic absorption spectrophotometry. Mercury was measured with a LEEMAN Hydra AA mercury analyser. The oil in water was measured with a Varian Cary Eclipse Fluorescence Spectrophotometer. The salinity in water was measured with a Huguang (Shanghai, China) SYC1–2 salinity meter.

### Statistical analysis

One-way analysis of variance (ANOVA) and linear regressions analysis with the “Enter” or “Stepwise” method were performed using SPSS 20.0 software. Results with *p* values of less than 0.05 were considered statistically significant.

## Additional Information

**How to cite this article**: Fan, L. *et al*. Spatial distribution of planktonic bacterial and archaeal communities in the upper section of the tidal reach in Yangtze River. *Sci. Rep.* **6**, 39147; doi: 10.1038/srep39147 (2016).

**Publisher's note:** Springer Nature remains neutral with regard to jurisdictional claims in published maps and institutional affiliations.

## Supplementary Material

Supplementary Table S1

## Figures and Tables

**Table 1:** Richness (the Chao index) and diversity (the Shannon index) of bacterial and archaeal communities.

	Anqing	Tongling	Wuhu	Nanjing	Jingjiang
Bacteria					
The Chao index value	808.33 ± 46.19	764.33 ± 58.23	834.33 ± 69.58	844.33 ± 26.10	861.00 ± 36.04
The Shannon index value	4.67 ± 0.06	4.39 ± 0.46	4.59 ± 0.27	4.72 ± 0.18	4.36 ± 0.31
Archaea					
The Chao index value	457.33 ± 33.62	450.67 ± 141.73	662.67 ± 301.62	541.00 ± 81.46	1059.00 ± 105.53
The Shannon index value	5.10 ± 0.24	4.97 ± 0.60	5.40 ± 0.49	5.42 ± 0.17	5.28 ± 0.86

**Table 2:** Correlation coefficients and significance values for Linear Regressions of distances from the mouth of estuary versus the relative abundance related to functional taxa of bacterial and archaeal communities.

Functional description (dependent variables)	Bacteria		Archaea	
	P value	R^2^	P value	R^2^
A: RNA processing and modification	0.095	0.199	0.492	0.037
B: Chromatin structure and dynamics	0.009*	0.418	0.604	0.021
C: Energy production and conversion	0.022*	0.342	0.468	0.041
D: Cell cycle control, cell division, chromosome partitioning	0.036*	0.296	0.718	0.01
E: Amino acid transport and metabolism	0.017*	0.364	0.332	0.072
F: Nucleotide transport and metabolism	0.055	0.255	0.439	0.047
G: Carbohydrate transport and metabolism	0.081	0.216	0.087	0.209
H: Coenzyme transport and metabolism	0.045*	0.275	0.686	0.013
I: Lipid transport and metabolism	0.024*	0.335	0.090	0.205
J: Translation, ribosomal structure and biogenesis	0.028*	0.319	0.511	0.034
K: Transcription	0.040*	0.285	0.165	0.143
L: Replication, recombination and repair	0.059	0.248	0.624	0.019
M: Cell wall/membrane/envelope biogenesis	0.023*	0.336	0.158	0.470
N: Cell motility	0.006*	0.455	0.579	0.024
O: Posttranslational modification, protein turnover, chaperones	0.012*	0.093	0.213	0.117
P: Inorganic ion transport and metabolism	0.016*	0.370	0.276	0.090
Q: Secondary metabolites biosynthesis, transport and catabolism	0.032*	0.307	0.089	0.206
R: General function prediction only	0.034*	0.342	0.358	0.065
S: Function unknown	0.013*	0.390	0.455	0.044
T: Signal transduction mechanisms	0.010*	0.413	0.164	0.144
U: Intracellular trafficking, secretion, and vesicular transport	0.006*	0.449	0.372	0.062
V: Defense mechanisms	0.179	0.134	0.297	0.083
W: Extracellular structures	0.190	0.128	0.504	0.035
Z: Cytoskeleton	0.007*	0.442	0.000*	0.651

**Table 3:** Linear Regressions of distances from the mouth of estuary (independent variables) versus environmental variables and the responded environmental variables (independent variables) versus the first NMDS dimensions of bacterial and archaeal similarities.

Distance		Environmental variables	Bacteria		Archaea	
P value	R^2^		P value	R^2^	P value	R^2^
0.045*	0.274	Nitrate	0.368	0.068	0.001*	0.586
0.014*	0.381	Sulfate	0.000*	0.664	0.017*	0.389
0.020*	0.351	Ammonia	0.563	0.028	0.521	0.038
0.006*	0.405	Cadmium	0.697	0.013	0.680	0.016
0.002*	0.540	Lead	0.420	0.055	0.640	0.021
0.015*	0.376	Turbidity	0.196	0.135	0.693	0.015
0.044*	0.276	Chlorine	0.504	0.038	0.381	0.070
0.026*	0.326	Bromine	0.408	0.058	0.817	0.005
0.069	0.233	Temperature				
0.636	0.018	Total nitrogen				
0.605	0.021	Nitrite				
0.639	0.017	Total phosphorus				
0.102	0.013	Permanganate index				
0.116	0.179	Chlorophyll a				
0.423	0.050	Zinc				
0.158	0.147	Mercury				
0.494	0.037	Arsenic				
0.169	0.140	Copper				
0.257	0.058	Oil				
0.886	0.002	Fluorine				
0.985	0.000	TOC				
0.560	0.027	TC				
0.231	0.018	IC				
