# Supplementary material for: Spatial distribution of planktonic bacterial and archaeal communities in the upper section of the tidal reach in Yangtze River
Source: Sci Rep. 2016 Dec 14;6:39147. doi: 10.1038/srep39147 (PMC5155431; doi:10.1038/srep39147)
Supplement: Supplementary Table S1 [file srep39147-s1.pdf]

Spatial distribution of planktonic bacterial and archaeal communities in the upper section of the tidal reach in Yangtze River

Limin Fan[1,+], Chao Song[1,+], Shunlong Meng[1], Liping Qiu[1], Yao Zheng[1], Wei Wu[1], Jianhong Qu[1],Dandan Li[1], Cong Zhang[1],Gengdong Hu[1*] &Jiazhang Chen[1*]

1 Freshwater Fisheries Research Center, Chinese Academy of Fishery Sciences, Scientific Observing and Experimental Station of Fishery Resources and Environment in the Lower Reaches of the Yangtze River, Wuxi 214081, China

*corresponding authors:
J Z Chen: E-mail: chenjz@ffrc.cn
G D ·Hu: E-mail: hugd@ffrc.cn

+ these authors contribute equally to this work

Table.S1 The geographical position information (longitude and latitude coordinate) of the sampling sites

The sampling and the reference sites	Longitude and latitude coordinate	The distance between the sampling sites and Dongwangsha (km)
Anqing	N 30.62° E 117.23°	619.55
Tongling	N 30.82° E 117.72°	566.45
Wuhu	N 31.34° E 118.35°	443.75
Nanjing	N 32.09° E118.73°	348.85
Jingjiang	N 31.95° E 120.13°	171.15
